# Comparative mitogenomics of the Decapoda reveals evolutionary heterogeneity in architecture and composition

**DOI:** 10.1038/s41598-019-47145-0

**Published:** 2019-07-24

**Authors:** Mun Hua Tan, Han Ming Gan, Yin Peng Lee, Heather Bracken-Grissom, Tin-Yam Chan, Adam D. Miller, Christopher M. Austin

**Affiliations:** 1 Centre of Integrative Ecology, School of Life and Environmental Sciences Deakin University, Geelong, Australia; 2 Deakin Genomics Centre, Deakin University, Geelong, Australia; 3 Genomics Facility, Tropical Medicine and Biology Platform, Monash University Malaysia, Jalan Lagoon Selatan, Bandar Sunway 47500, Petaling Jaya, Selangor Malaysia; 4 School of Science, Monash University Malaysia, Jalan Lagoon Selatan, Bandar Sunway 47500, Petaling Jaya, Selangor Malaysia; 5 Department of Biological Sciences, Florida International University, North Miami, Florida 33181 USA; 6 Institute of Marine Biology and Center of Excellence for the Oceans, National Taiwan Ocean University, 2 Pei-Ning Road, Keelung, 20224 Taiwan

**Keywords:** Phylogenetics, Data processing, Molecular evolution

## Abstract

The emergence of cost-effective and rapid sequencing approaches has resulted in an exponential rise in the number of mitogenomes on public databases in recent years, providing greater opportunity for undertaking large-scale comparative genomic and systematic research. Nonetheless, current datasets predominately come from small and disconnected studies on a limited number of related species, introducing sampling biases and impeding research of broad taxonomic relevance. This study contributes 21 crustacean mitogenomes from several under-represented decapod infraorders including Polychelida and Stenopodidea, which are used in combination with 225 mitogenomes available on NCBI to investigate decapod mitogenome diversity and phylogeny. An overview of mitochondrial gene orders (MGOs) reveals a high level of genomic variability within the Decapoda, with a large number of MGOs deviating from the ancestral arthropod ground pattern and unevenly distributed among infraorders. Despite the substantial morphological and ecological variation among decapods, there was limited evidence for correlations between gene rearrangement events and species ecology or lineage specific nucleotide substitution rates. Within a phylogenetic context, predicted scenarios of rearrangements show some MGOs to be informative synapomorphies for some taxonomic groups providing strong independent support for phylogenetic relationships. Additional comparisons for a range of mitogenomic features including nucleotide composition, strand asymmetry, unassigned regions and codon usage indicate several clade-specific trends that are of evolutionary and ecological interest.

## Introduction

Traditional molecular systematic research has been largely dependent on phylogenetic reconstructions based on few molecular markers obtained via individual targeted polymerase chain reaction (PCR) and Sanger sequencing approaches^1–7^. More recently, the advent of second-generation sequencing (SGS), coupled with the development of simplified and streamlined laboratory and bioinformatic pipelines^8–12^, has seen a proliferation of eukaryotic and prokaryotic genomic resources. Notably, advances in genomic approaches have greatly increased the efficiency of recovering complete and near-complete mitochondrial genomes from species across the animal and plant kingdoms, making it a popular candidate for phylogenetic and comparative genomic studies, in addition to its role as a useful barcoding locus for species identification^13^. Despite the rapid growth in mitogenome resources, current datasets predominately come from small and disconnected studies on single or a limited number of related species, introducing sampling biases and impeding research of broad taxonomic relevance^14–16^. While there is a general consensus that new mitogenome resources remain valuable for comparative genomic and systematic research, improved representation of mitogenome resources for poorly-represented biological lineages is still required for studies aimed at addressing fundamental evolutionary, genomic and systematic questions^14^. More specifically, within the context of phylogenetics, inadequate taxon sampling and taxon biases can lead to topological distortions due to artefactual sources of error such as long-branch attraction^17^. This highlights the need for reviews of sampling biases and more exhaustive sampling to overcome such limitations.

Decapods are a highly speciose and diverse group of crustaceans, including 11 infraorders and almost 15,000 species^18^, which provide critical ecosystem services across marine, freshwater and terrestrial environments, and are of significant commercial and/or medical importance^19–22^. The first decapod mitogenome sequences emerged in the early 2000s based on tedious Sanger sequencing approaches but were biased primarily toward species of commercial importance^23–28^. While these studies provide an early hint on the heterogeneous nature of decapod mitogenomes, reporting novel gene orders in multiple lineages, phylogenetic and comparative genomic studies remained severely constrained by limited taxon sampling for almost a decade^23–28^. However, since the emergence of modern genomic sequencing approaches, the availability of mitogenome resources for the Decapoda has rapidly expanded. After the study by Miller and colleagues^24,25^, the number of decapod mitogenomes has expanded rapidly (now numbering in the hundreds), proving a valuable resource for studies that have provided new insights into decapod evolutionary relationships and comparative mitogenomics at various taxonomic levels^11,29–36^. Despite this progress, sampling within this large and diverse group has generally been skewed towards commercial or easily-sampled species, highlighting the need for more balanced taxonomic sampling^17,36–38^. At the time this study was initiated, mitogenomic resources for the less diverse and rare groups such as Polychelida, Stenopodidea, Axiidea and Gebiidea were scarce, which impeded the resolution of their relative positions within previous mitogenome phylogenetic reconstructions^35,36^. To gain a more comprehensive understanding of evolutionary relationships and genomic compositions within Decapoda, mitogenome resources for more species across a breadth of taxonomic groups are much needed.

Though the value of mitogenomes as the sole genetic marker for phylogenetic research is often contested^6,39,40^, mitochondrial DNA sequences remain the most abundant publicly-available molecular genetic resources, which continue to facilitate fundamental evolutionary research^11,35,36^. As mitogenomic resources continue to expand and bioinformatic tools capable of processing and analysing increasingly immense datasets are further developed, opportunities for more comprehensive analyses of mitogenome structural architecture and systematic relationships across broad taxonomic groups are emerging^14,15^. This is giving rise to increasing numbers of studies on the evolution of mitogenome features across various animal groups^35,41,42^. Specifically, these new resources are allowing transcriptional and translational features such as nucleotide and amino acid composition^43,44^, substitution rates^34,45^, codon usage^32,46^, unassigned and intergenic regions^47,48^ and strand asymmetry^49,50^ to be contrasted within and among a range of taxonomic groups.

As a whole, lineages within the Decapoda have successfully conquered a very broad range of environments (e.g. freshwater, terrestrial, marine) as well as adopted a wide variety of lifestyles, body forms and sizes. Considering the varied metabolic demands associated with this range of adaptations, it is expected that respiratory functions of the mitochondrion are subject to substantial selective pressures^51,52^. Thus, heterogeneity in mitogenomic features may provide insights into possible correlations between the evolution of the molecule and species diversification at a range of evolutionary scales^41,53^. The ease of recovering complete mitogenomes with modern genomic approaches is now allowing for the analysis of genomic structural diversification in unprecedented detail^42,47,54–56^. In the Decapoda, novel mitochondrial gene orders (MGOs) have been reported across a range of lineages^57–61^ and at an unusually high frequency compared with other higher-level taxa (e.g. several insect orders^62^, frogs^63^, birds^49^, batoids^64^, elasmobranchs^65^). Several studies have suggested possible correlations of certain highly-rearranged MGOs to accelerated nucleotide substitution rates in selected lineages or to the adaptations to specialised lifestyles and extreme ecological niches^34,45,62,66^. This may potentially be multifactorial, occurring in association with the evolution of various biochemical and metabolic traits (e.g. DNA repair, metabolic rate, generation time, body size)^45^. Similar to insects^62^, MGO patterns have been identified as potential synapomorphies for various decapod groups at a range of taxonomic levels, in some instances providing unequivocal resolution of contentious evolutionary relationships^31,32,34,66–69^.

In this study, we contribute 21 new mitogenomes from 7 decapod infraorders including a number from pre-identified taxonomic groups with limited sampling. We provide an overview of the distribution of mitochondrial gene orders (MGO) within the context of our inferred phylogeny and show that these rearrangements are unevenly distributed throughout Decapoda. Though noting a lack of correlation of these heightened rearrangements with variations in general ecology, lifestyles or nucleotide substitution rates, we point out instances of MGOs as likely synapomorphies at various taxonomic levels and also included comparisons of several mitogenomic features to gain further insights into decapod mitogenome evolution and phylogeny.

## Results

### New mitogenomes for under-represented infraorders

This work contributes mitogenomes for 21 species from 7 decapod infraorders (Table 1), substantially increasing complete mitogenome resources for infraorders that were most under-represented in previous mitogenome studies. All but one of the mitogenomes were successfully assembled into complete circular sequences with sizes ranging from 15.5 to 17.6 kbp. The assembled mitogenome of *Parastacus brasiliensis* contains a gap in the control region. All mitogenomes contain the typical 13 protein-coding genes and 2 ribosomal RNAs (rRNA), but with varying numbers (18 to 23) of identified transfer RNAs (tRNA) (Supplementary Data S1).

**Table 1:** Specimen sources and mitogenome accession numbers for 21 decapod species (from 7 infraorders) contributed in this study.

Family	Species	Location	Specimen voucher	Accession number	Raw read SRA
**ACHELATA**					
Scyllaridae	*Ibacus alticrenatus*	Off Ningaloo North, Western Australia, Australia	NMV J53370	MG551493	SRR7698955
Scyllaridae	*Remiarctus bertholdii*	Off Ningaloo South, Western Australia, Australia	NMV J53384	MG551497	SRR7698953
Palinuridae	*Puerulus angulatus*	Off Ningaloo North, Western Australia, Australia	NMV J55596	MG551496	SRR7698954
**ASTACIDEA**					
Parastacidae	*Ombrastacoides huonensis*	West of Scotts Peak Road near Twin Creek, Tasmania, Australia	N/A	MG551494	SRR7698936
Parastacidae	*Parastacus brasiliensis*	Brazil	N/A	MG551495	SRR7698933
**AXIIDEA**					
Strahlaxiidae	*Strahlaxius plectrorhynchus*	Edithburg, Yorke Peninsula, South Australia, Australia	AM P82855	MH234571	SRR7698923
**CARIDEA**					
Alpheidae	*Alpheus inopinatus*	Casuarina beach, Darwin, Northern Territory, Australia	N/A	MG551491	SRR7698912
**GEBIIDEA**					
Axianassidae	*Axianassa australis*	Praia do Araçá, São Sebastião, São Paulo, Brazil	NMV J44613	MH234568	SRR7698901
Laomediidae	*Laomedia healyi*	Merimbula Lake, New South Wales, Australia	AM P41482	MH234569	SRR7698907
Thalassinidae	*Thalassina squamifera*	Rapid Creek, Darwin, Northern Territory, Australia	N/A	MG551498	SRR7698904
Upogebiidae	*Gebiacantha plantae*	Off Yorke Island, Queensland, Australia	NMV J44914	MG551492	SRR7698906
Upogebiidae	*Upogebia affinis*	Sandbank on south side of Fort Pierce inlet, Florida, USA	NMV J40668	MH234572	SRR7698905
Upogebiidae	*Upogebia bowerbankii*	Off Ningaloo North, Western Australia, Australia	NMV J53465	MG551499	SRR7698908
**POLYCHELIDA**					
Polychelidae	*Cardus crucifer*	Golfe ibéro-marocain (35.19N, 7.84W)	MNHN IU-2008-10482	KX343003	SRR7698881
Polychelidae	*Pentacheles laevis*	South China Sea (10.32N, 114.23E)	NTOU M01875	KX343004	SRR7698884
Polychelidae	*Polycheles baccatus*	Arafura Sea, Northern Territory, Australia	MAGNT Cr006219	MH234570	SRR7698883
Polychelidae	*Polycheles coccifer*	Donggang Fishing Port, Southwest Taiwan	NTOU M01757	KX343005	SRR7698886
Polychelidae	*Stereomastis sculpta*	Northern Gulf of Mexico, Louisiana, USA	N/A	KX343002	SRR7698885
**STENOPODIDEA**					
Spongicolidae	*Globospongicola spinulatus*	Southwest Taiwan (22.27N, 120.00E)	NTOU M01877	KU188326	SRR7698888
Spongicolidae	*Spongicola levigatus*	South China Sea (16.12N, 114.34E)	NTOU M01876	KU188325	SRR7698887
Stenopodidae	*Stenopus scutellatus*	Carrie Bow Cay, Belize	N/A	MF741653	SRR7698889

### Mitogenome-based phylogenetics provide insights into infra-ordinal relationships

**Figure 1:**
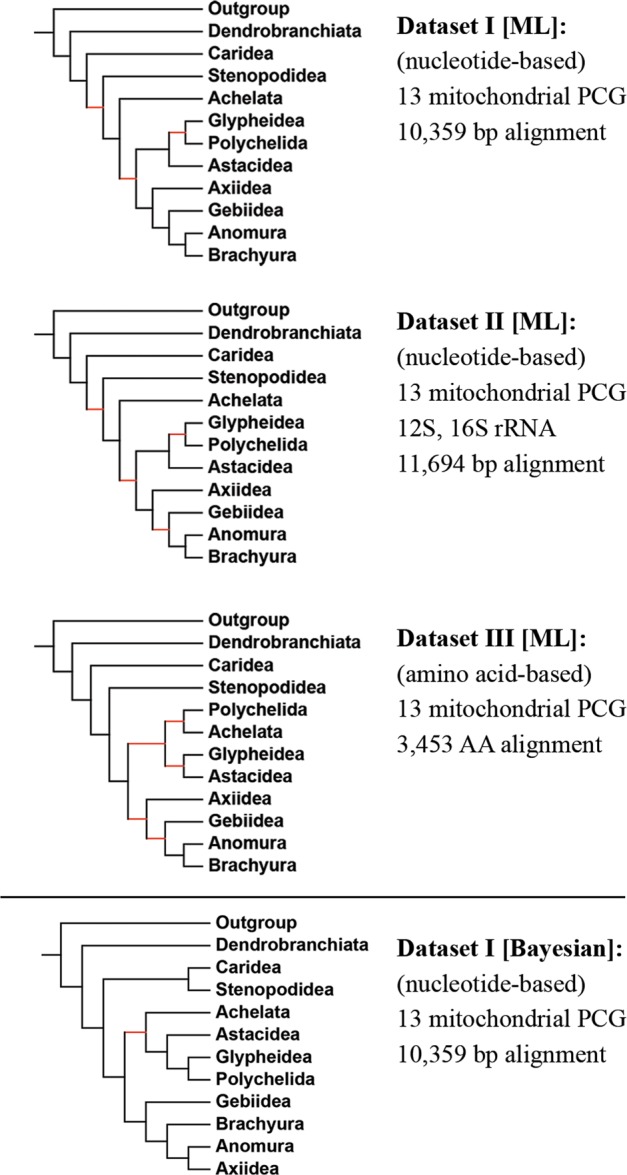
Infraorder-level topology inferred from Maximum likelihood (ML) and Bayesian (BI) methods, based on three datasets. Red branches indicate nodes with weak support (ML: ultrafast bootstrap values of < 95%, BI: posterior probabilities of <0.90).

Concatenation of the mitochondrial genes resulted in 10 359, 11 694 and 3 453 alignment sites for Dataset I (nucleotide-based, 13 PCG), Dataset II (nucleotide-based, 13 PCG + 2 rRNA) and Dataset III (amino acid-based, 13 PCG), respectively. Out of the 21 new species for which mitogenomes were contributed in this study, eight species are observed in the trees to share sister relationships with species from the same genus. The remaining 13 species represent new lineages, providing new resources for their respective genera (*Spongicola, Globospongicola, Remiarctus, Puerulus, Cardus, Pentacheles, Stereomastis, Parastacus, Ombrastacoides, Strahlaxius, Axianassa, Laomedia, Gebiacantha*). These lineages also cluster with other members from their respective families or superfamilies supporting existing taxonomic arrangements (Fig. 1, trees are available as Supplementary Data S2). Relationships among all decapod infraorders are represented in this analysis, with the exception of Procarididea that lacks available complete mitogenome data at the time of this study.

All three datasets recover monophyletic groups for the 10 Pleocyemata infraorders, with a consistent basal position for the shrimps (Caridea, Stenopodidea), followed by the burrowing shrimps (Axiidea, Gebiidea), with the ‘crabs’ (Anomura, Brachyura) in the most derived positions. The lobsters and crayfish and allies, mostly represented by Achelata, Astacidea, Glypheidea and Polychelida, see some discrepancies between topologies reconstructed from nucleotide (Dataset I and II) and amino acid (Dataset III) datasets. Nucleotide-based phylogenies place Achelata at a relatively basal position whereas the topology based on the amino acid alignment has Achelata nested within the larger lobster/crayfish clade. This, in turn, affects the positions of the other lobster/crayfish infraorders as well. On the other hand, the positions of several infraorders are variable in the Bayesian-inferred tree (Fig. 1), resulting in a clade of Caridea + Stenopodidea sharing a sister relationship. Also in this tree, while Axiidea and Gebiidea are still recovered as separate infraorders, the latter is placed at a more basal position, an observation that contrasts with the topologies shown in the other three trees.

### Gene order rearrangements are unevenly distributed across decapod infraorders

**Figure 2:**
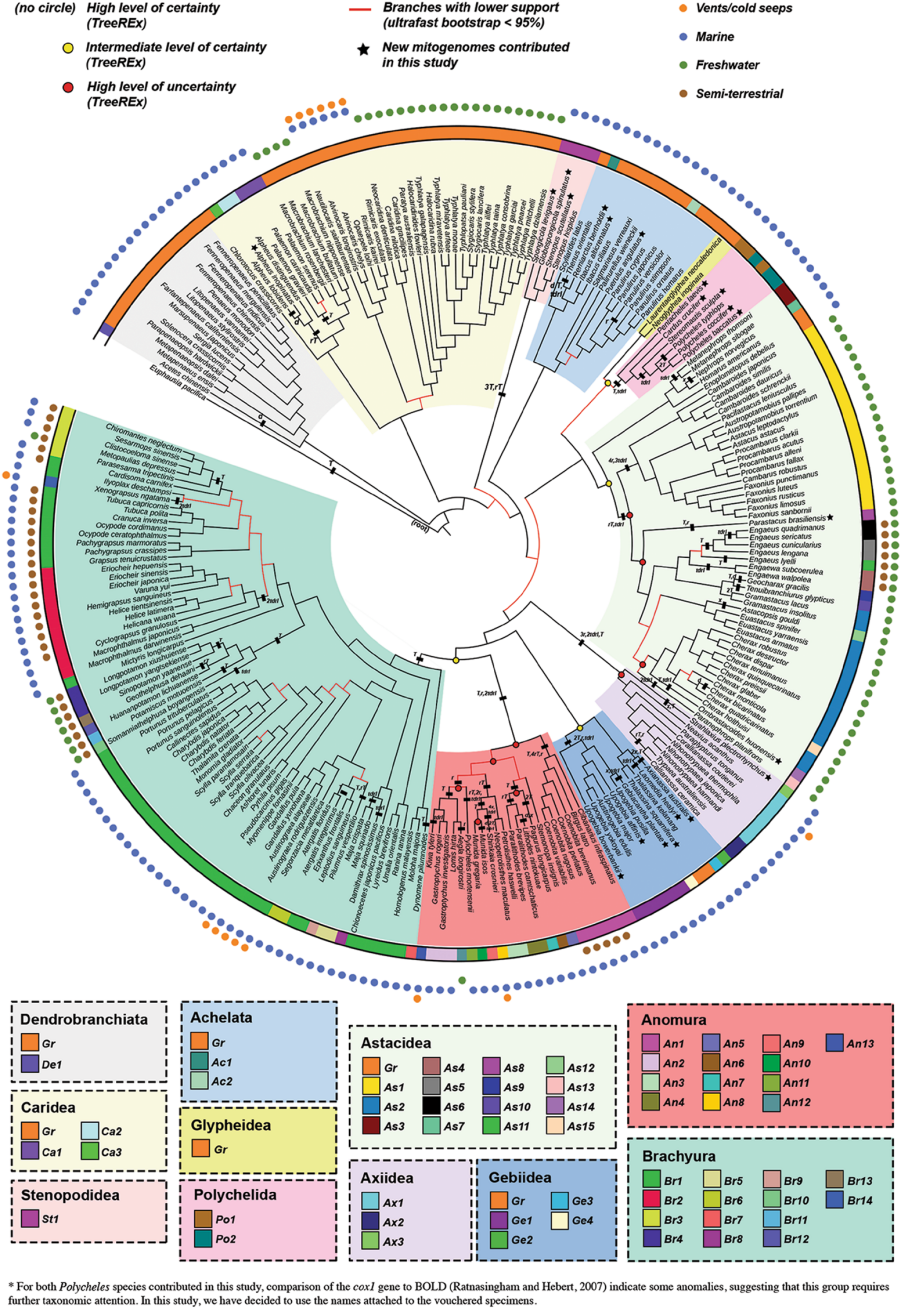
Decapod phylogenetic tree. This cladogram was inferred using the maximum-likelihood method based on Dataset I (13 mitochondrial PCGs, 10 359 nucleotide alignment). Clades are coloured according to the different infraorders. The outer colour strip in the phylogenetic tree represents the distribution of mitochondrial gene orders (MGO) in various infraorders and summarises a total of 59 different MGOs across the 246 different decapod species analysed, labelled for each infraorder in the panels below. Orange-coloured MGO labelled with ‘*Gr*’ refers to the pancrustacean ground pattern; other derived MGOs are numerically labelled and attached with a 2-letter infraorder prefix. MGOs that differ from the ground pattern are a result of a series of CREx-predicted gene rearrangement events: transposition (*T*), reversal (*r*), reverse transposition (*rT*), duplication (*d*), deletion (*x*) and tandem duplication-random loss (*tdrl*). Yellow- or red-coloured circles on some nodes reflect the level of uncertainty for the TreeREx reconstruction of each ancestral MGO, with red exhibiting highest level of uncertainty, yellow for mid-level and no circle for consistent reconstruction (see Babbucci, *et al*.^42^ for details). Subsequent outer rings indicate, to the best of our knowledge, the possible environments (terrestrial, freshwater, marine, vents/seeps) inhabited by each decapod species.

In general, the level of diversity in MGO patterns varies widely within and between infraorders (Fig. 2). Excluding the infraorders with less than 10 species sampled (Glypheidea, Stenopodidea, Polychelida, Axiidea), decapod groups ordered from most to least diversity of MGOs are as follows: Anomura (13 MGO patterns among 22 species), Gebiidea, Astacidea, Achelata, Brachyura, Dendrobranchiata and Caridea (4 MGO patterns among 37 species). Several MGO ‘hot-spots’ are identified from this analysis. For instance, almost every family in Anomura (*An*) and Gebiidea (*Ge*) has a unique MGO pattern. Within the Astacidea (*As*), species in the Parastacidae exhibit 12 different MGOs compared to its sister family group Astacidae with only one MGO pattern shared by all species. In addition, almost every genus in the brachyuran (*Br*) superfamilies Gecarcinucoidea and Potamoidea have unique MGOs (see Supplementary Data S3 for additional information).

Established as a gene order common to insects and crustaceans^67^, the pancrustacean ground pattern (coloured orange and labelled *Gr*) is predominantly observed for basal decapod groups such as the suborder Dendrobranchiata and infraorders Caridea, Achelata and Glypheidea, appears in a limited number of species within the Astacidea and Gebiidea, and is absent in all species sequenced so far in the other infraorders. Within the context of the inferred phylogeny, gene rearrangement events shared by members of specific clades provide examples of unifying evolutionary signatures (synapomorphies) from higher levels such as the infraorder-specific *St1* pattern shared by the four analysed Stenopodidea species, through the superfamily-specific *As1* for species in the Astacoidea, and family-specific rearrangements at the base of the Upogebiidae clade illustrated by the *Ge1* pattern.

### Lack of correlation between MGO variation and substitution rates

Overall, evidence of episodic positive selection is observed in 10 out of 13 mitochondrial PCGs (*ATP6, COX1, COX2, COX3, CYTB, ND1, ND2, ND4, ND5, ND6*) and for main branches of most infraorders/suborders, with the exception of Dendrobranchiata, Glypheidea and Axiidea (Supplementary Data S4). Notably, a higher frequency of statistically significant selection is reported in Caridea mostly detected along branches leading to the genera *Alpheus*, *Palaemon* and *Macrobrachium*. On the other hand, accelerated evolutionary rates are observed for multiple decapod lineages across all infraorders (Supplementary Data S4). Based on observation, while there appears to be a link between substitution rates and heightened gene rearrangements in some taxonomic clades, for example in Parastacoidea (southern hemisphere crayfish) or Caridea, there are also instances where accelerated rates are observed for lineages with highly conserved MGOs as well. However, across Decapoda as a whole, the Spearman correlation analysis does not suggest a link between variable MGOs and nucleotide/amino acid substitution rates (Supplementary Data S4).

### Mitogenome characteristics vary within and between decapod infraorders

**Figure 3:**
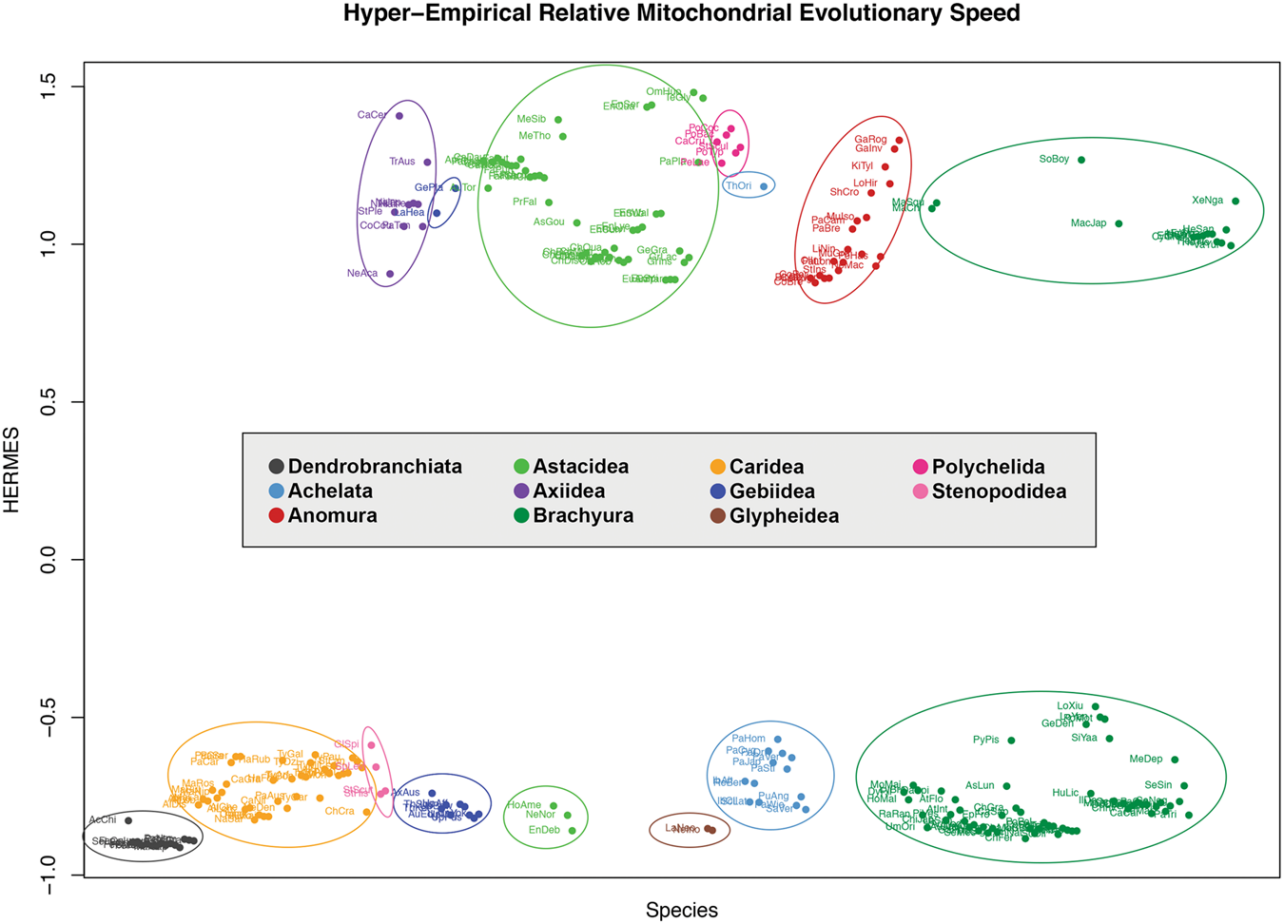
HERMES index across Decapoda. Species are listed horizontally by suborder, infraorder, superfamilies and families to highlight differences among various taxonomic groups.

Applying the Hyper-Empirical Relative Mitochondrial Evolutionary Speed (HERMES) index developed by Plazzi, *et al*.^41^, the amount of mitochondrial evolution was estimated based on several genomic features found to be phylogenetically congruent in their tested datasets. These genomic features include the root-to-tip distance for each species (RtoTdist), ML distance from the *E. pacifica* outgroup (MLdist), percentage of Unassigned Regions (URs), amount of mitochondrial identical gene arrangements (AMIGA) for PCGs and strand usage skew (SUskew). The HERMES index appears to be informative for our dataset with reasonable goodness-of-fit statistics: Tucker-Lewis Index (TLI)^70^ = 0.939; root mean square of the residuals (SRMR) = 0.053; root mean squared error of approximation (RMSEA) = 0.058, all close to boundaries suggested by Hu and Bentler^71^. The highest communality was for AMIGA at 85.5% whereas other variables scored at only 10 to 20%, resulting in a mean of 29.1% (i.e. the HERMES index accounts for 29.1% of the total variability of the source matrix). Since gene order is the key parameter in this system, the HERMES factor analysis in Fig. 3 separates individuals into two distinct groups. The first group exhibits low HERMES index and consists of individuals with MGO that is identical to or highly similar to the pancrustacean ground pattern (*Gr*) while the second group comprises mostly of individuals that have undergone *tdrl* events resulting in large rearrangements of mitochondrial PCGs (Fig. 2).

**Figure 4:**
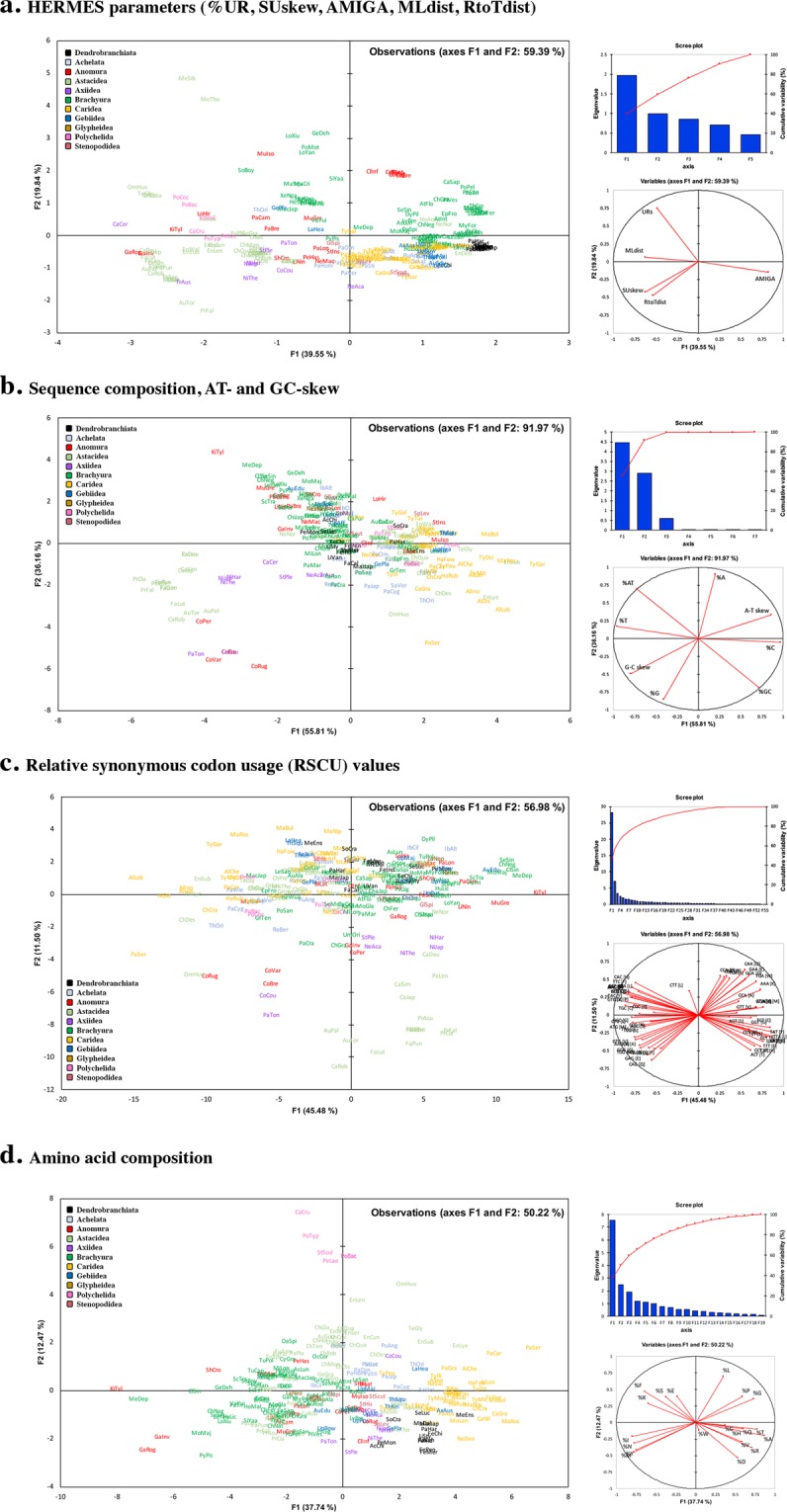
Mitogenomic PCA plots. Principal component analyses using Pearson’s correlation based on various characteristics of the mitogenome. (**a**) shows the PCA plot based on the same five variables in the HERMES analysis, with the first two principal components accounting for 59.39% of the dataset variability. Additionally, (**b**–**d**) summarise nucleotide composition, asymmetry (skew) information and amino acid composition with 91.97%, 56.98% and 56.98% of each dataset variability, respectively, in the first two principal components. Data points are labelled with the first 2 or 3 letters of the genus followed by the first 3 letters of the species name; e.g. ‘*ChDes*’ for *Cherax destructor*.

Several notable observations can be made on the clustering of data points based on various mitogenomic features, as observed in principal component analysis (PCA) plots in Fig. 4. The plot in Fig. 4a is based on the same five variables in the previous HERMES analysis. While most individuals with MGOs identical or similar to the pancrustacean ground pattern tend to form a tight cluster, some of these are distinctly separated from others based on the proportion of their unassigned regions, most of them either containing more than one control region (e.g. *Metanephrops*, *Munida*) or long intergenic regions (e.g. *Geothelphusa*, *Longpotamon*) (Fig. 4a). Additionally, Fig. 4b–d summarise nucleotide composition, asymmetry (skew) information and amino acid composition respectively. The AT content separates most of the brachyuran crabs from caridean shrimps, whereas GC-skew distinguishes the two Astacidea superfamilies (Astacoidea, Parastacoidea) (Fig. 4b). The two burrowing mud shrimp infraorders, Axiidea and Gebiidea, are often clearly split into different quadrants for plots based on nucleotide features, whereas data points representing anomuran species are generally widely scattered across all PCA plots.

## Discussion

This study adds to the growing list that has benefited from the use of museum-preserved specimens for mitogenomic studies^37,72–77^, with mitogenomes for 21 species strategically sampled to fill important taxonomic gaps, particularly in under-represented decapod infraorders. The higher level topologies of all three Maximum likelihood trees reconstructed in this study are congruent at the base and top of the trees (Fig. 1), consistently recovering: [1] The suborder Dendrobranchiata as sister group to the rest of egg-brooding Pleocyemata, [2] Caridea as basal to Pleocyemata, [3] Stenopodidea as sister to all of Reptantia, [4] Axiidea and Gebiidea as separate infraorders, and [5] Anomura and Brachyura placed as sister taxa high in the tree, generally in agreement with previous reports^1,11,36,37^ though with points of distinction with others^2,3,6,78,79^. While the higher level topology of the Bayesian-inferred tree generally exhibits similar observations, there are deviations with respect to the relationship between Caridea and Stenopodidea (recovered as sister clades) and the shuffle in positions of Axiidea, Anomura and Brachyura. Generally, incongruent topologies show that inter-relationships among lobster and crayfish infraorders still remain unresolved^1,2,6,7,78,80,81^, requiring further taxonomic sampling and/or additional nuclear gene information. It is also noteworthy that the taxonomically-impoverished infraorder Procarididea^5,82^ is absent from our study, but is considered by most to likely be one of the basal lineages within the Decapoda^5^.

The phylogenetic analysis presented in this study represents one of the most comprehensive samplings of decapod species, based on mitochondrial data. While genome-based phylogenies are still rare for the Decapoda due to the generally large sizes of its genomes (1 to 40 Gbp), the increasingly lower costs of sequencing are enabling studies to target more genomic loci through genome skimming or anchored hybrid enrichment methods^83–85^. However, these still lack the fine-grained resolution obtained in this study. Two noteworthy studies that have been recently published have attempted to elucidate decapod inter-relationships based on genomic (Wolfe, *et al*.^84^; 94 decapod species, 410 loci, greater than 86 000 bp) or transcriptomic data (Schwentner, *et al*.^86^; 16 decapod species, 81 to 684 orthogroups, 17 690 to 242 530 amino acid positions). Mitogenome-based maximum likelihood phylogenies in this study share similarities in parts of their topologies with that obtained from these studies, specifically in the recovery of shrimp and prawn groups as basal clades and the traditional Meiura (Brachyura + Anomura) as more derived lineages, increasing the confidence of these nodes based on support from different underlying genetic data. Nevertheless, several nodes are still in contention. This study recovers a paraphyletic Caridea and Stenopodidea clade whereas both Wolfe, *et al*.^84^ and Schwentner, *et al*.^86^ recovered these infraorders as sister clades. The position of Achelata also remains unresolved in most trees as well as the interrelationships among crayfish and lobster groups, as well as the positions of the traditional thalassinids (Axiidea, Gebiidea). Overall, topologies inferred in this study are generally more similar to that obtained by Wolfe, *et al*.^84^.

The contrasting MGO patterns across all available decapod mitogenomes have revealed some interesting findings. Most notably, a number of highly-rearranged gene orders are observed, occurring in unequal frequencies across various decapod infraorders or at lower taxonomic levels, with a level of diversity higher than or at least comparable to the levels observed in other metazoan groups^42,47,49,63,87,88^. We see the prevalence of the pancrustacean ground pattern (*Gr*)^67^, and/or other highly-similar MGOs in infraorders at both the basal and more derived positions, indicating that this highly-conserved ground pattern is likely to be ancestral for decapods, with modified MGOs within each infraorder being more derived traits^35^. Hence, instances of clade-specific MGO patterns or MGO “hot spots” suggest that the utility of MGOs as synapomorphies is amplified for certain decapod groups, acting as unifying evolutionary signatures that can be used to either support or question existing classifications at a range of taxonomic levels^31,32,34,35,62,66,67,69^. For example, brachyuran species associated with the Varunidae (superfamily Grapsoidea) and Macrophthalmidae (superfamily Ocypodoidea) that form sister clades^31,66,89,90^ also share the *Br2* MGO pattern, which provides complementary support to other phylogenetic studies that call for a re-evaluation of classifications for the two large paraphyletic superfamilies^33,89–91^. Nonetheless, it is equally important that we continue to be cautious when evaluating MGO information within a phylogenetic context^42,66,92^, keeping in mind the caveats related to potential homoplastic or convergent arrangements^42,87,93,94^, a saturation of signals from reversible rearrangement events^95,96^ or possibly inaccurate inferences from unresolved phylogenies^66^.

In contrast to findings that found a positive correlation between gene rearrangements and elevated nucleotide substitution rates in insect and bivalve mitogenomes^41,45^, our correlation analysis indicates no such association for the Decapoda (Spearman correlation in Supplementary Data S4). Although the exact drivers of MGO rearrangements remain unclear, other studies have noted broad associations with adaptations to extreme ecological niches and lifestyles^34,62,66,68,97,98^. Similarly, accelerated rates of MGO rearrangements occur in various decapod lineages such as burrowing crayfish^34^, freshwater crabs^66^, and other species adapted to extreme deep-sea environments or temperatures^66,98^. However, again, our study indicates no such correlation between MGOs and ecologically-similar decapod groups. For example, freshwater crabs and crayfish (southern hemisphere parastacids) display highly variable MGOs, while other freshwater groups such as the shrimps and northern hemisphere crayfish maintain the same gene order among all sampled species (although the members of northern hemisphere crayfish group have a large distinctive rearrangement that acts as a synapomorphy for the superfamily). The same observation applies to other crab and shrimp species inhabiting deep sea vent niches, or burrowing axiid mud shrimps, which exhibit only a few rearranged MGOs.

However, ecological transitions and the evolution of a distinct Bauplan or the adoption of different lifestyles (e.g. parasitism or adaptation to freshwater) may be achieved via different evolutionary pathways^99–101^ and the consequences for energy demands for an organism may be just one of a plethora of factors including thermal tolerance^102^, aerial exposure^103^, sensitivity to salinity^104^, light^105^ or metal concentrations^106^. In addition to the regulation of their transcription and translation^107,108^, mitochondria are very dynamic structures often undergoing continuous fusion, fission and motility, events that can potentially affect their bioenergetic capacities^109^. However, the extent of these rearrangements and the cues that trigger them are still poorly understood. Thus, we do not rule out an association of heightened MGO rearrangements and profound changes at deep taxonomic levels (e.g. the two groups of crayfish) to clade-specific adaptations and ecological/life history transitions, but these cannot be confidently determined until we achieve a better understanding of the stressors involved at the cellular and physiological level and the associated adaptive responses.

Previous studies have compared decapod MGO patterns at lower taxonomic levels across the Decapoda^31,32,34,35,60,66,110,111^. However, this study is one of the few to investigate the extent of MGO diversity at the ordinal level and a large number of species (246 species), the other being a phylomitogenomic study on 86 malacostracan mitogenomes^38^. Other large-scale comparative MGO analyses for various metazoan groups have also reported on the distribution of MGOs, some showing relatively high rates of MGO evolution from their respective ancestral patterns. These examples include not only invertebrate groups such as insects, gastropods, lice, barnacles, bivalves and annelids^42,56,62,112–114^, but also vertebrates such as in fish, frogs, salamanders and amphisbaenian lizards^87,115–118^. Conversely, there are other studies that show high levels of conservatism across major taxonomic groups such as beetles, echinoids, batoids and elasmobranchs^64,65,119,120^. While decapod mitogenomes clearly showcase a high diversity of MGO patterns as a whole (59 MGOs across 246 species), it is premature to claim that this degree of diversity is unusual or comparable to that of other metazoan groups since results are influenced by the number of mitogenomes compared (i.e. scale of comparison), the taxonomic level of interest or as a result of unbalanced or biased sampling. We therefore highly recommend interpreting the findings from any comparative analyses with caution especially those still lacking representation for major groups and again, urge for more consideration to be given to better sampling strategies.

Comprehensive studies on the evolutionary trends of mitogenomes (both small and large scale) are also emerging for various animal groups, following the increased availability of mitogenomic resources^41,119,121–126^. To determine if there are other aspects of the decapod mitogenome that may show clade-specific associations, aside from the arrangement of genes, we compared additional aspects of the molecular architecture and composition of mitogenomes across the major decapod groups. Mitogenomes analysed in this study are generally around 16 kbp in length, though some outliers were identified (17–20 kbp) with lengths inflated by a higher proportion of unassigned regions, i.e. containing more than one control region (e.g. *Metanephrops* species^61^) or relatively long stretches of intergenic spaces such as in most Potamoidea species^127,128^. Though proportions of unassigned regions may potentially be the discerning feature for some taxonomic groups^129^, this notion should also be treated with caution as the sizes of these regions may be influenced by the assembly or annotation methods used to predict genes, exemplified by the identification of three *trnL* genes by Segawa and Aotsuka^127^ as opposed to only two copies reported following re-annotations by MITOS in this study^10^. But generally, decapod mitogenome sizes are relatively stable compared to those of other metazoan groups such as bivalves, nematodes and sponges that exhibit strong heterogeneity in size^88^, with some larger molecules spanning lengths of over 20 kbp and even up to 48 kbp^130^.

Strand and nucleotide composition asymmetries are other mitochondrial characteristics that have been noted to vary across animal groups^49,50,131–133^. In this study, we point out observed trends from PCA plots that suggest molecular signatures for certain decapod groups. Similar to insects, annelids and arachnids, decapod mitogenomes are generally AT-rich, as opposed to lower compositional AT bias often observed in chordates (e.g. fishes, birds, reptiles)^134^. Within the Decapoda itself, recognizable clusters were observed for Caridea (average: 65%, range: 59% to 70%) and Brachyura (average: 71%, range: 65% to 77%) with minor overlaps. Other noteworthy features include positive GC-skew values that appear to be a unifying characteristic for all northern hemisphere freshwater crayfish (Astacoidea) as well as for the four *Coenobita* anomuran species included in this study, as opposed to negative GC-skew values for most other decapods. Further, species from infraorders Axiidea and Gebiidea are well separated due to substantial differences in compositional bias and asymmetry, which, in addition to previous reports from phylogenetic and MGO analyses^6,35,135,136^, is consistent with their status as separate infraorders instead of united in what was once the Thalassinidea^1,78,137,138^. On the other hand, data points for anomuran species are dispersed across all plots, highlighting their higher plasticity and mitogenomic variability within this infraorder notorious for its morphological and ecological diversity, including the convergent evolution of the “crab” form^66,69,101,139,140^ and taxonomic controversies^141–144^.

For all discussed groups with their own unique signatures, it is of interest to see if these patterns still hold when more mitogenomes are included in future analyses. Though this study is limited in scope to comparisons across whole mitogenomes, we recognise that this only skims the surface of the range of possible compositional comparisons (e.g. at the gene level) and that there are a myriad of other factors that can be included in further detailed investigations, e.g. testing different codon sites^43,44^ or the effects of these asymmetries on nucleotide versus amino acid compositions^50,145^.

## Methods

### Sequencing, mitogenome assembly and annotation

Most samples used for this study were from vouchered specimens obtained from museum collections, including the National Taiwan Ocean University (NTOU), Museum Victoria (NMV), Muséum National d’Histoire Naturelle (MNHN), Australian Museum (AM) and Museum and Art Gallery of the Northern Territory (MAGNT). Using the Sokolov method^146^, genomic DNA was extracted from the tissue samples of 21 crustacean species (Table 1) representing seven decapod infraorders (Gebiidea: 6, Polychelida: 5, Achelata: 3, Stenopodidea: 3, Astacidea: 2, Axiidea: 1, Caridea: 1), processed using Nextera-based library preparation (Illumina, USA) and sequenced at low coverage on the Illumina MiSeq platform located at the Monash University Malaysia Genomics Facility to generate paired-end short reads (2 × 250 bp), as previously described^8^. Low quality sequences were trimmed with Trimmomatic v.0.36^147^ (*illuminaclip*:2:30:10, *avgqual*:20, *leading*:3, *trailing*:3, *minlen*:50), followed by contaminant-filtering (i.e. sequences of bacterial origin) with kraken v.0.10.5-beta^148^ using its precompiled database (minikraken_20141208). Resulting high-quality reads were then assembled *de novo* with IDBA-UD v.1.1.1^149^ to recover a complete mitogenome or, if circularity was not obtained, MITObim v.1.8^9^ was used to achieve assemblies using short mtDNA sequences from related species as ‘baits’. Circularised mitogenomes were annotated with MITOS^10^ to identify gene boundaries for protein-coding genes (PCG), ribosomal RNAs (rRNA) and transfer RNAs (tRNA), which were further adjusted manually based on sequence homology to genes available on NCBI/GenBank^150^.

### Mitogenome-based phylogenetics

Based on combinations of protein-coding genes (PCG) and ribosomal RNA genes (16S, 12S), mitogenome sequences from 246 decapod species, with *Euphausia pacifica* (Euphausiacea) as the outgroup, maximum-likelihood trees were constructed with the MitoPhAST v3.0 pipeline^11^, which automates sequence alignments, trimming of ambiguous regions, sequence concatenation, partitioning by gene (and codon position for nucleotides), model testing and tree-building by combining a series of tools^151–158^. Analyses were carried out on three datasets:

I. 13 mitochondrial PCGs (nucleotides).
II. 13 mitochondrial PCGs + 12 S + 16 S (nucleotides).
III. 13 mitochondrial PCGs (amino acids).

All Maximum-likelihood trees reconstructed from the three mitochondrial-based datasets (Datasets I to III) were rooted with *E. pacifica* (Euphausiacea), which is often treated as sister clade to the Decapoda^159^. Support at each node is evaluated with 1000 ultrafast bootstrap replicates (UFBoot)^160^ and 1000 SH-aLRT replicates (SH)^161^.

### Mitochondrial gene order (MGO) analysis

Prior to gene order analyses, public mitogenome entries downloaded from GenBank were manually inspected for inaccuracies via cross-checks against results from the MITOS webserver^10^. Any discrepancies found (e.g. incorrect strands specified, missing genes, mislabelling, etc) were corrected and new GenBank files were generated (Supplementary Data S5). Mitogenomes were then processed through the MitoPhAST v3.0 pipeline, which compares and clusters MGOs into groups according to gene order patterns^66^. Similar to Tan, *et al*.^66^, the output Fasta format file containing MGO for every mitogenome was pre-processed to remove missing genes in one or more species and also to retain only a single copy of duplicated genes. Using each of the generated Maximum-likelihood trees (nucleotide- and amino acid-based) as a phylogenetic guide, gene re-arrangement pathways and putative ancestral MGOs were reconstructed with TreeREx v.1.85^54^. To obtain more accurate inferences, TreeREx results were further used to guide pairwise comparisons using web-based CREx^55^ to enable the inclusion of fuller sets of genes that would have been excluded in the TreeREx analysis. The interactive Tree of Life (iTOL) online phylogenetic tool^162^ was used to illustrate the distribution of MGOs across various infraorders in the phylogenetic tree.

### Comparison analyses of other mitochondrial features

Excluding individuals with partial mitogenomes, Hyper-Empirical Relative Mitochondrial Evolutionary Speed (HERMES) index is generated for each of 239 individuals using HERMES v.1.0^41^ to estimate the amount of mitochondrial evolution. Given a maximum likelihood tree, the program computes the root-to-tip distance for each species (RtoTdist) and ML distance from the *E. pacifica* outgroup (MLdist) then merges these with other mitochondrial characteristics including the percentage of Unassigned Regions (URs), amount of mitochondrial identical gene arrangements (AMIGA) for PCGs and strand usage skew (SUskew). Also, codon usage counts in each individual were obtained with EMBOSS v.6.6.0^163^ with minor adjustments for the Invertebrate Mitochondrial Code, followed by the calculation of relative synonymous codon usage (RSCU) values by taking the ratio of the actual number of times a codon appears to the expected frequency of the codon if all synonymous codons for the same amino acid are used equally^164^. Various mitogenome characteristics were then summarised by Principal Component Analysis (PCA) using Pearson correlation carried out with XLSTAT v.2018.5.52460^165^.

### Episodic selection, evolutionary rates and spearman correlation test

Without making an a priori assumption on the likelihood of specific lineages undergoing episodic positive selection, the aBSREL method^166^ implemented in the command-line version of the HyPhy v.2.3.11 package^167^ was used on the codon alignment of each mitochondrial protein-coding gene to test each branch in the maximum -likelihood phylogeny inferred from Dataset I, to inspect whether a proportion of sites have evolved under positive selection (ω > 1). Further, evolutionary rates were estimated from the same codon alignments using BEAST v.2.5.0^168^. An uncorrelated, lognormal relaxed clock model was applied to each partition and the Yule Model was used as the tree prior since taxa consist of individuals from different species, with *Euphausia pacifica* set as outgroup. Two BEAST MCMC runs of 6 × 10^9^ were performed and convergence was checked with Tracer v.1.7.1^169^, applying a burn-in of 20% and checking for sufficient Effective Sample Size (ESS) (>200). Finally, trees from both MCMC runs were combined and a maximum clade credibility tree was constructed. Rates were visualised with Figtree v.1.4.3^170^. Correlations among different MGOs, habitat and substitution rates were measured by the Spearman correlation coefficient carried out with XLSTAT v.2018.5.52460^165^, details in Supplementary Data S4.

## Conclusion

Moving forward, further research is needed to contrast the composition and architecture of mitogenomes across other metazoan groups to determine if our observed trends are common or specific to the Decapoda. As more mitogenomic data is made available, coupled with bioinformatics tools such as the MGO analysis feature in the MitoPhAST pipeline, it becomes increasingly feasible to conduct more complete and larger-scale comparisons for any animal group of interest in the future, the bottleneck now being to ensure that annotations in public database entries are accurate, which in this study was still a manual process. We have been fortunate to benefit from mitogenomes generated by various research groups that have contributed to enriching these resources for the order Decapoda but also realise that there is still need for new mitogenomes, carefully sampled to achieve a more balanced representation of infraorders. More importantly, by demonstrating what is possible for large-scale comparative MGO analyses in this study, it is our hope to inspire the undertaking of future research equivalent to or surpassing the extent of this study, contributing as a collective to the eventual reformation of the field of mitochondrial genomics.

## Supplementary information

Supplementary Data S1

Supplementary Data S2

Supplementary Data S3

Supplementary Data S4

Supplementary Data S5

## Data Availability

Assembled mitogenomes are available on NCBI repository (See Table 1 for GenBank SRA accession numbers). Raw datasets generated during and/or analysed during the current study are available from the corresponding author on reasonable request.
