# Supplementary material for: Comparative mitogenomics of the Decapoda reveals evolutionary heterogeneity in architecture and composition
Source: Sci Rep. 2019 Jul 24;9:10756. doi: 10.1038/s41598-019-47145-0 (PMC6656734; doi:10.1038/s41598-019-47145-0)
Supplement: Supplementary file 4 — Supplementary Data S4 [file 41598_2019_47145_MOESM4_ESM.zip › Supplementary_Data_S4.pdf]

Supplementary Data S4

Comparative mitogenomics of the Decapoda reveals evolutionary heterogeneity in architecture and composition

MUN HUA TAN*, HAN MING GAN, YIN PENG LEE, HEATHER BRACKEN-GRISSOM, TIN-YAM CHAN, ADAM D. MILLER, CHRISTOPHER M. AUSTIN

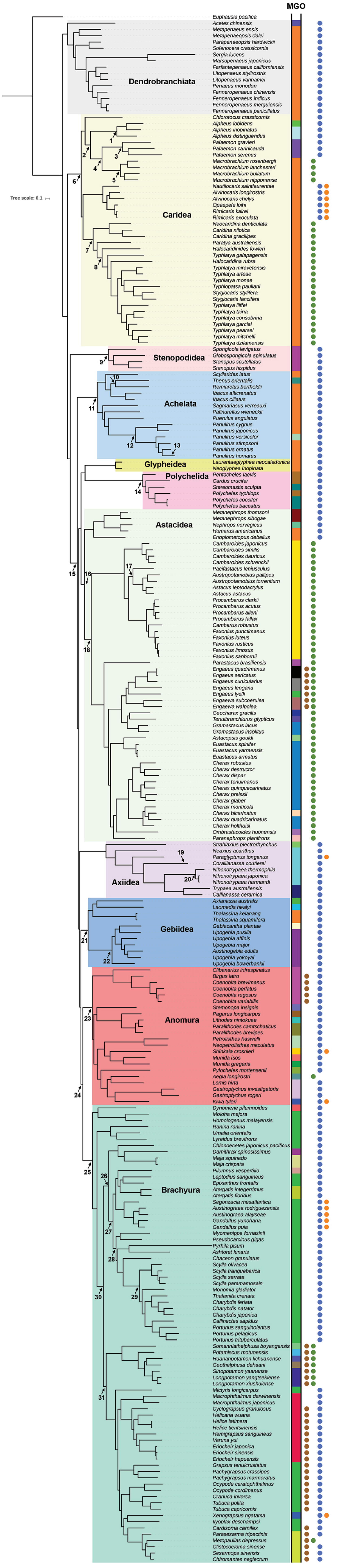

Episodic diversifying selection detected by HyPhy (aBSREL)

[significance assessed with Likelihood Ratio Test at a threshold of p ≤ 0.05, after correcting for multiple testing]

B	Optimized branch length
LRT	Likelihood ratio test statistic for selection
Test p-value	P-value corrected for multiple testing
ω_2 distribution	Inferred ω estimates and respective proportion of sites

Node label	Gene	B	LRT	Test p-value	ω_2 distribution
1	ATP6	0.6649	21.9604	0.0028	101 (7.8%)
	ND5	0.6649	27.9469	0.0001	72.1 (9.7%)
2	ND2	0.1353	18.5544	0.0152	899 (6.7%)
3	COX1	0.5163	24.6300	0.0007	3330 (2.3%)
4	COX2	0.2899	20.9719	0.0046	6370 (7.5%)
	ND1	0.2899	19.3592	0.0102	895 (3.4%)
	ND5	0.2899	21.8030	0.0030	10000 (6.0%)
5	ND5	0.4609	17.0507	0.0321	670 (6.4%)
6	ND2	0.3439	60.2270	0.0000	8740 (18%)
	ND4	0.3439	18.1430	0.0187	3330 (5.4%)
	ND6	0.3439	21.5706	0.0034	432 (7.9%)
7	ATP6	0.2537	18.3231	0.0171	10000 (3.8%)
	COX1	0.2537	18.0185	0.0201	210 (2.8%)
	CYTB	0.2537	17.6088	0.0247	10000 (1.9%)
	ND2	0.2537	21.4234	0.0036	140 (14%)
	ND5	0.2537	24.6624	0.0007	331 (8.4%)
8	ND4	0.1287	25.6557	0.0004	10000 (4.9%)
9	ATP6	0.9529	17.3206	0.0283	91.2 (14%)
	ND5	0.9529	53.0387	0.0000	678 (8.2%)
10	COX1	0.1181	17.5021	0.0259	676 (1.4%)
11	ATP6	0.5007	20.8533	0.0048	1240 (10%)
	ND2	0.5007	19.5309	0.0094	215 (14%)
12	CYTB	0.6800	22.6406	0.0020	162 (8.4%)
13	ND1	0.3104	33.5453	0.0000	1640 (10%)
14	ND4	1.4463	25.0377	0.0006	85.6 (14%)
	ND5	1.4463	18.5484	0.0151	10000 (11%)
15	ND5	0.1753	53.0719	0.0000	6060 (7.1%)
16	ATP6	0.0842	20.8438	0.0048	494 (6.4%)
	COX1	0.0842	27.1618	0.0002	8640 (1.5%)
17	ND2	0.1065	16.2870	0.0475	469 (4.5%)
18	ND5	0.1719	21.6181	0.0033	10000 (2.9%)
19	ND2	1.1062	19.4981	0.0095	21.5 (23%)
20	ATP6	0.7905	19.1513	0.0113	1350 (12%)
21	CYTB	0.1795	20.4981	0.0058	194 (2.8%)
22	ND4	0.6004	20.2131	0.0066	44.7 (11%)
23	COX3	0.2354	17.0931	0.0321	116 (4.1%)
	ND4	0.2354	27.2521	0.0002	356 (6.7%)
24	ND1	0.0495	21.9636	0.0028	452 (2.8%)
25	ND1	0.2430	17.1163	0.0314	659 (3.6%)
	ND4	0.2430	18.7054	0.0141	175 (5.6%)
	ND5	0.2430	23.8897	0.0010	750 (4.8%)
26	ND1	0.1311	17.9246	0.0210	10000 (3.3%)
	ND4	0.1311	31.8677	0.0000	10000 (5.6%)
	ND5	0.1311	19.5682	0.0091	508 (3.3%)
27	ND2	0.1101	20.0817	0.0071	10000 (6.3%)
28	ND1	0.0761	19.7990	0.0082	3330 (2.4%)
29	ND4	0.4172	19.9706	0.0075	97.8 (10%)
	ND5	0.4172	27.4205	0.0002	10000 (6.6%)
30	ATP6	0.1637	22.0514	0.0027	10000 (4.2%)
	COX1	0.1637	17.7753	0.0226	7170 (0.72%)
	ND5	0.1637	32.0589	0.0000	10000 (3.8%)
31	ND2	0.0715	20.0539	0.0072	96.3 (7.9%)

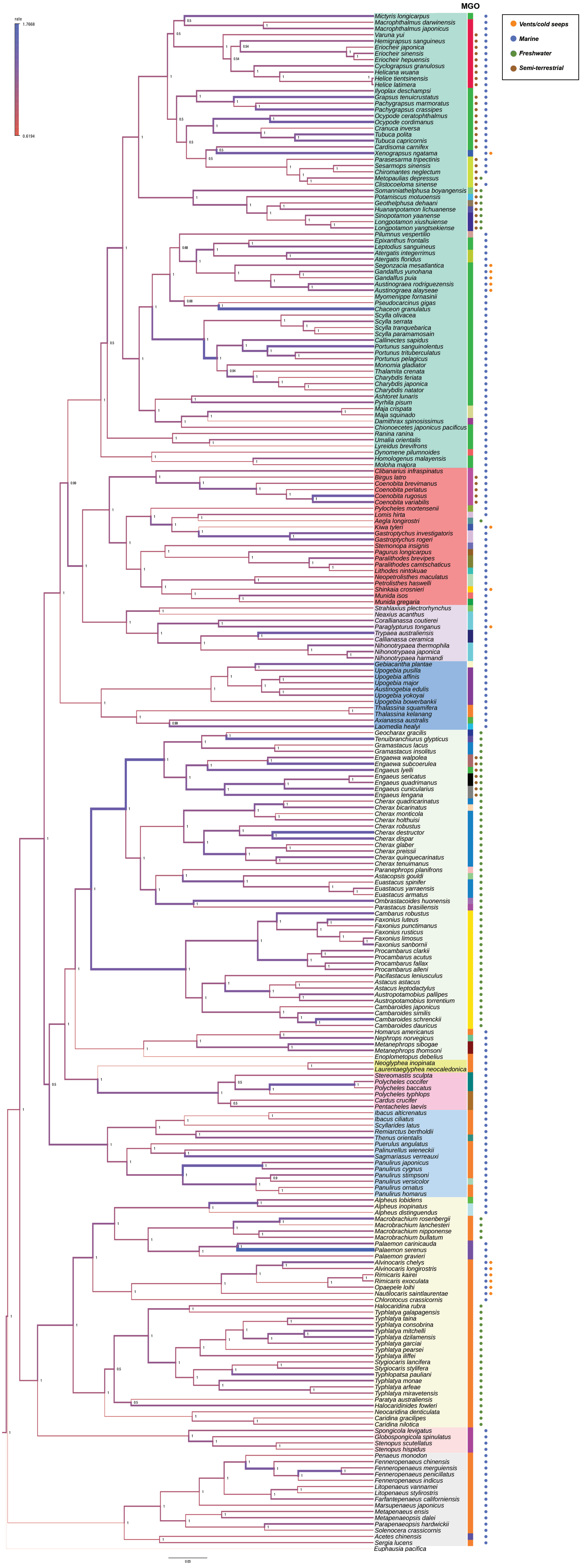

MGO
rate
1.7668
0.6194
Vents/cold seeps
Marine
Freshwater
Semi-terrestrial
Mictyris longicarpus
Macrophthalmus darwinensis
Macrophthalmus japonicus
Varuna yui
Hemigrapsus sanguineus
Eriocheir japonica
Eriocheir sinensis
Eriocheir hepuensis
Cyclograpsus granulosus
Helicana wuana
Helice tientsinensis
Helice latimera
Ilyoplax deschampsi
Grapsus tenuicrustatus
Pachygrapsus marmoratus
Pachygrapsus crassipes
Ocypode ceratophthalmus
Ocypode cordimanus
Cranuca inversa
Tubuca polita
Tubuca capricornis
Cardisoma carnifex
Xenograpsus ngatama
Parasesarma tripectinis
Sesarmops sinensis
Chiromantes neglectum
Metopaulias depressus
Clistocoeloma sinense
Somanniathelphusa boyangensis
Potamiscus motuoensis
Geothelphusa dehaani
Huananpotamon lichuanense
Sinopotamon yaanense
Longpotamon xiushuiense
Longpotamon yangtsekiense
Pilumnus vespertilio
Epixanthus frontalis
Leptodius sanguineus
Atergatis integerrimus
Atergatis floridus
Segonzacia mesatlantica
Gandalfus yunohana
Gandalfus puia
Austinograea rodriguezensis
Austinograea alayseae
Myomenippe fornasinii
Pseudocarcinus gigas
Chaceon granulatus
Scylla olivacea
Scylla serrata
Scylla tranquebarica
Scylla paramamosain
Callinectes sapidus
Portunus sanguinolentus
Portunus trituberculatus
Portunus pelagicus
Monomia gladiator
Thalamita crenata
Charybdis feriata
Charybdis japonica
Charybdis natator
Ashtoret lunaris
Pyrhila pisum
Maja crispata
Maja squinado
Damithrax spinosissimus
Chionoecetes japonicus pacificus
Ranina ranina
Umalia orientalis
Lyreidus brevifrons
Dynomene pilumnoides
Homologenus malayensis
Moloha majora
Clibanarius infraspinatus
Birgus latro
Coenobita brevimanus
Coenobita perlatus
Coenobita rugosus
Coenobita variabilis
Pylocheles mortensenii
Lomis hirta
Aegla longirostri
Kiwa tyleri
Gastroptychus investigatoris
Gastroptychus rogeri
Stemonopa insignis
Pagurus longicarpus
Paralithodes brevipes
Paralithodes camtschaticus
Lithodes nintokuae
Neopetrolisthes maculatus
Petrolisthes haswelli
Shinkaia crosnieri
Munida isos
Munida gregaria
Strahlaxius plectrorhynchus
Neaxius acanthus
Corallianassa coutierei
Paraglypturus tonganus
Trypaea australiensis
Callianassa ceramica
Nihonotrypaea thermophila
Nihonotrypaea japonica
Nihonotrypaea harmandi
Gebiacantha plantae
Upogebia pusilla
Upogebia affinis
Upogebia major
Austinogebia edulis
Upogebia yokoyai
Upogebia bowerbankii
Thalassina squamifera
Thalassina kelanang
Axianassa australis
Laomedia healyi
Geocharax gracilis
Tenuibranchiurus glypticus
Gramastacus lacus
Gramastacus insolitus
Engaewa walpolea
Engaewa subcoerulea
Engaeus lyelli
Engaeus sericatus
Engaeus quadrimanus
Engaeus cunicularius
Engaeus lengana
Cherax quadricarinatus
Cherax bicarinatus
Cherax monticola
Cherax holthuisi
Cherax robustus
Cherax destructor
Cherax dispar
Cherax glaber
Cherax preissii
Cherax quinquecarinatus
Cherax tenuimanus
Paranephrops planifrons
Astacopsis gouldi
Euastacus spinifer
Euastacus yarraensis
Euastacus armatus
Ombrastacoides huonensis
Parastacus brasiliensis
Cambarus robustus
Faxonius luteus
Faxonius punctimanus
Faxonius rusticus
Faxonius limosus
Faxonius sanbornii
Procambarus clarkii
Procambarus acutus
Procambarus fallax
Procambarus alleni
Pacifastacus leniusculus
Astacus astacus
Astacus leptodactylus
Austropotamobius pallipes
Austropotamobius torrentium
Cambaroides japonicus
Cambaroides similis
Cambaroides schrenckii
Cambaroides dauricus
Homarus americanus
Nephrops norvegicus
Metanephrops sibogae
Metanephrops thomsoni
Enoplometopus debelius
Neoglyphea inopinata
Laurentaeglyphea neocaledonica
Stereomastis sculpta
Polycheles coccifer
Polycheles baccatus
Polycheles typhlops
Cardus crucifer
Pentacheles laevis
Ibacus alticrenatus
Ibacus ciliatus
Scyllarides latus
Remiarctus bertholdii
Thenus orientalis
Puerulus angulatus
Palinurellus wieneckii
Sagmariasus verreauxi
Panulirus japonicus
Panulirus cygnus
Panulirus stimpsoni
Panulirus versicolor
Panulirus ornatus
Panulirus homarus
Alpheus lobidens
Alpheus inopinatus
Alpheus distinguendus
Macrobrachium rosenbergii
Macrobrachium lanchesteri
Macrobrachium nipponense
Macrobrachium bullatum
Palaemon carinicauda
Palaemon serenus
Palaemon gravieri
Alvinocaris chelys
Alvinocaris longirostris
Rimicaris kairei
Rimicaris exoculata
Opaepele loihi
Nautilocaris saintlaurentae
Chlorotocus crassicornis
Halocaridina rubra
Typhlatya galapagensis
Typhlatya taina
Typhlatya consobrina
Typhlatya mitchelli
Typhlatya dzilamensis
Typhlatya garciai
Typhlatya pearsei
Typhlatya iliffei
Stygiocaris lancifera
Stygiocaris stylifera
Typhlopatsa pauliani
Typhlatya monae
Typhlatya arfeae
Typhlatya miravetensis
Paratya australiensis
Halocaridinides fowleri
Neocaridina denticulata
Caridina gracilipes
Caridina nilotica
Spongicola levigatus
Globospongicola spinulatus
Stenopus scutellatus
Stenopus hispidus
Penaeus monodon
Fenneropenaeus chinensis
Fenneropenaeus merguiensis
Fenneropenaeus penicillatus
Fenneropenaeus indicus
Litopenaeus vannamei
Litopenaeus stylirostris
Farfantepenaeus californiensis
Marsupenaeus japonicus
Metapenaeus ensis
Metapenaeopsis dalei
Parapenaeopsis hardwickii
Solenocera crassicornis
Acetes chinensis
Sergia lucens
Euphausia pacifica
0.03

Correlation matrix (Spearman):

Variables	AMIGA	NT_RtoDist	AA_RtoDist	habitat
AMIGA	**1**	0.024	0.013	-0.035
NT_RtoDist	0.024	**1**	**0.938**	-0.033
AA_RtoDist	0.013	**0.938**	**1**	0.028
habitat	-0.035	-0.033	0.028	**1**

Values in bold are different from 0 with a significance level alpha=0.05

p-values (Spearman):

Variables	AMIGA	NT_RtoDist	AA_RtoDist	habitat
AMIGA	**0**	0.707	0.846	0.593
NT_RtoDist	0.707	**0**	**< 0.0001**	0.607
AA_RtoDist	0.846	**< 0.0001**	**0**	0.672
habitat	0.593	0.607	0.672	**0**

Coefficients of determination (Spearman):

Variables	AMIGA	NT_RtoDist	AA_RtoDist	habitat
AMIGA	**1**	0.001	0.000	0.001
NT_RtoDist	0.001	**1**	0.879	0.001
AA_RtoDist	0.000	0.879	**1**	0.001
habitat	0.001	0.001	0.001	**1**

Correlation maps:

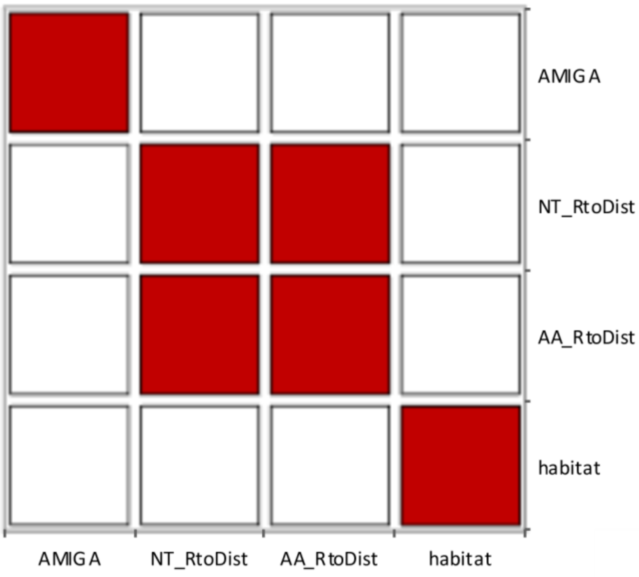

Input data:

ID	AMIGA	NT_RtoDist	AA_RtoDist	habitat
AcChi	0	3.885	1.324	2
SeLuc	0.261603	5.921	1.166	2
FaCal	0.261603	3.643	1.189	2
FeChi	0.261603	3.661	1.179	2
FeInd	0.261603	3.714	1.174	2
FeMer	0.261603	3.698	1.177	2
FePen	0.261603	3.703	1.178	2
LiSty	0.261603	3.681	1.167	2
LiVan	0.261603	3.677	1.159	2
MarJap	0.261603	4.917	1.147	2
MeDal	0.261603	3.531	1.171	2
MeEns	0.261603	3.502	1.159	2
PaHar	0.261603	3.51	1.142	2
PeMon	0.261603	3.788	1.217	2
SoCra	0.261603	3.575	1.181	2
AlDis	0.00421941	4.184	1.48	2
AlIno	0.00421941	4.29	1.487	2
AlLob	0	4.345	1.526	2
AlLon	0.261603	3.736	1.267	5
AlChe	0.261603	3.855	1.297	5
NaSai	0.261603	3.711	1.228	5
OpLoi	0.261603	3.684	1.252	5
RiExo	0.261603	3.673	1.243	5
RiKai	0.261603	3.675	1.243	5
CaGra	0.261603	4.385	1.528	3
CaNil	0.261603	4.057	1.397	3
HaRub	0.261603	4.571	1.668	3
HaFow	0.261603	4.273	1.542	3
NeDen	0.261603	4.018	1.368	3
PaAus	0.261603	4.026	1.363	3
StLan	0.261603	4.682	1.667	3
StSty	0.261603	4.61	1.643	3
TyArf	0.261603	4.328	1.551	3
TyCon	0.261603	4.348	1.588	3
TyDzi	0.261603	4.468	1.619	3
TyGal	0.261603	4.368	1.577	3
TyGar	0.261603	4.19	1.518	3
TyIli	0.261603	4.478	1.575	3
TyMir	0.261603	4.317	1.558	3
TyMit	0.261603	4.469	1.61	3

Habitat codes	
1	Vents/cold seep
2	Marine
3	Freshwater
4	Semi-terrestrial
5	Seeps + marine
6	Freshwater + Semi-terrestrial
7	Marine + Semi-terrestrial

TyMon	0.261603	4.474	1.559	3
TyPea	0.261603	4.563	1.616	3
TyTai	0.261603	4.382	1.587	3
TyPau	0.261603	4.776	1.698	3
MaBul	0.261603	4.236	1.535	3
MaLan	0.261603	4.243	1.461	3
MaNip	0.261603	4.195	1.506	3
MaRos	0.261603	4.208	1.49	3
PaCar	0.00843882	4.619	1.684	2
PaGra	0.00843882	4.533	1.719	2
PaSer	0.00843882	4.824	1.661	2
ChCra	0.261603	3.808	1.287	2
GlSpi	0.0126582	4.254	1.463	2
SpLev	0.0126582	4.01	1.438	2
StHis	0.0126582	4.176	1.336	2
StScut	0.0126582	4.177	1.344	2
PaWie	0.261603	3.758	1.207	2
PaCyg	0.261603	4.574	1.498	2
PaHom	0.261603	4.637	1.537	2
PaJap	0.261603	4.485	1.455	2
PaOrn	0.261603	4.538	1.485	2
PaSti	0.261603	4.523	1.487	2
PaVer	0	4.392	1.474	2
PuAng	0.261603	3.84	1.233	2
SaVer	0.261603	3.706	1.221	2
IbAlt	0.261603	3.792	1.293	2
IbCil	0.261603	3.618	1.222	2
ReBer	0.261603	3.992	1.353	2
ScLat	0.261603	3.711	1.235	2
ThOri	0	3.993	1.342	2
LaNeo	0.261603	3.219	1.016	2
NeIno	0.261603	3.209	1.012	2
CaCru	0.00843882	4.451	1.598	2
PeLae	0.00843882	4.079	1.484	2
PoBac	0.00843882	4.407	1.549	2
PoCoc	0.00843882	4.404	1.544	2
PoTyp	0.00843882	4.464	1.572	2
StScul	0.00843882	4.31	1.476	2
AuPal	0.0590717	4.144	1.423	3
AuTor	0.0590717	4.073	1.395	3
PaLen	0.0590717	4.037	1.368	3
CaDau	0.0590717	4.046	1.34	3
CaJap	0.0590717	3.934	1.324	3

CaSim	0.0590717	4.05	1.359	3
CaRob	0.0590717	4.252	1.44	3
FaLim	0.0590717	4.187	1.406	3
FaLut	0.0590717	4.177	1.405	3
FaPun	0.0590717	4.152	1.392	3
FaRus	0.0590717	4.187	1.399	3
FaSan	0.0590717	4.188	1.406	3
PrAcu	0.0590717	4.174	1.417	3
PrCla	0.0590717	4.176	1.425	3
PrFal	0.0590717	4.165	1.419	3
EnDeb	0.261603	3.302	1.04	2
HoAme	0.261603	3.386	1.05	2
MeSib	0.00421941	3.455	1.12	2
MeTho	0.00421941	3.367	1.089	2
NeNor	0	3.491	1.063	2
AsGou	0	3.91	1.41	3
ChBic	0	3.982	1.45	3
ChDes	0.0590717	4.147	1.503	3
ChDis	0.0590717	4.094	1.478	3
ChGla	0.0590717	4.001	1.45	3
ChHol	0.0590717	4.064	1.431	3
ChMon	0.0590717	4.024	1.448	3
ChPre	0.0590717	4.044	1.459	3
ChQua	0.0590717	4.11	1.513	3
ChQui	0.0590717	3.997	1.446	3
ChRob	0.0590717	4.012	1.462	3
ChTen	0.0590717	4.019	1.445	3
EnCun	0.00421941	4.078	1.516	6
EnLen	0.00421941	4.199	1.574	6
EnLye	0	4.173	1.554	6
EnQua	0.00421941	4.077	1.528	6
EnSer	0.00421941	4.086	1.536	6
EnSub	0.00421941	4.21	1.655	6
EnWal	0.00421941	4.199	1.619	6
EuArm	0.0590717	3.693	1.337	3
EuSpi	0.0590717	3.707	1.337	3
EuYar	0.0590717	3.692	1.333	3
GeGra	0	3.895	1.434	3
GrIns	0.0590717	3.878	1.398	3
GrLac	0.0590717	3.836	1.398	3
OmHuo	0	4.094	1.534	3
PaPla	0	3.788	1.369	3
TeGly	0	4.049	1.552	3

CaCer	0.00421941	5.238	1.51	2
CoCou	0.021097	4.873	1.416	2
NiHar	0.021097	5.171	1.488	2
NiJap	0.021097	5.177	1.494	2
NiThe	0.021097	5.286	1.539	2
PaTon	0.021097	4.254	1.248	5
TrAus	0.00421941	5.432	1.513	2
NeAca	0.021097	4.071	1.17	2
StPle	0	4.3	1.279	2
AxAus	0	3.639	1.183	2
LaHea	0	3.656	1.172	2
ThKel	0.261603	3.498	1.145	2
ThSqu	0.261603	3.494	1.146	2
AuEdu	0.021097	3.655	1.087	2
GePla	0	3.606	1.083	2
UpAff	0.021097	3.504	1.06	2
UpBow	0.021097	3.436	1.059	2
UpMaj	0.021097	3.448	1.054	2
UpPus	0.021097	3.594	1.085	2
UpYok	0.021097	3.6	1.086	2
GaInv	0.00843882	4.406	1.397	2
GaRog	0.00843882	4.531	1.453	2
MuGre	0	3.704	1.173	2
MuIso	0	3.54	1.164	2
ShCro	0	3.71	1.318	5
NeMac	0.00421941	4.117	1.337	2
PeHas	0.00421941	4.145	1.345	2
StIns	0	3.386	1.068	2
KiTyl	0	3.792	1.213	5
LiNin	0	3.635	1.13	2
PaBre	0.00421941	3.613	1.114	2
PaCam	0.00421941	3.626	1.118	2
LoHir	0.00843882	3.447	1.076	2
BiLat	0.021097	3.544	1.093	7
CoBre	0.021097	3.926	1.104	7
CoPer	0.021097	4.116	1.136	7
CoRug	0.021097	4.134	1.14	7
CoVar	0.021097	4.056	1.136	7
ClInf	0.021097	3.736	1.167	2
PaLon	0	3.565	1.112	2
AuAla	0.172996	2.119	0.57	5
AuRod	0.172996	2.054	0.566	5
GaPui	0.172996	2.028	0.547	5

GaYun	0.172996	2.106	0.572	5
SeMes	0.172996	1.879	0.521	5
AsLun	0.172996	2.455	0.699	2
DyPil	0	2.853	0.889	2
MyFor	0.172996	2.008	0.524	2
PsGig	0.172996	1.791	0.47	2
EpFro	0.172996	2.005	0.525	2
SoBoy	0	2.814	0.849	6
CaCar	0.172996	2.498	0.709	7
GrTen	0.172996	2.888	0.835	7
PaCra	0.172996	2.913	0.813	7
PaMar	0.172996	2.859	0.81	7
ClSin	0.0168776	2.751	0.766	7
MeDep	0.0168776	2.966	0.906	6
SeNeg	0.0168776	2.73	0.743	7
SeSin	0.0168776	2.751	0.751	7
PaTri	0.0168776	2.672	0.766	7
CyGra	0.0379747	2.659	0.803	7
ErHep	0.0379747	2.904	0.833	7
ErJap	0.0379747	2.888	0.829	7
ErSin	0.0379747	2.903	0.83	7
HeWua	0.0379747	2.792	0.83	7
HeLat	0.0379747	2.757	0.814	7
HeTie	0.0379747	2.759	0.816	7
HeSan	0.0379747	2.878	0.852	7
VaYui	0.0379747	3.031	0.885	7
XeNga	0	2.983	0.855	5
HoMal	0.172996	3.013	0.935	2
MoMaj	0.172996	3.202	0.964	2
PyPis	0.172996	3.536	1.051	2
MaCri	0.00421941	2.584	0.719	2
MaSqu	0.00421941	2.6	0.735	2
DaSpi	0	2.844	0.814	2
ChiJap	0.172996	2.483	0.688	2
IlDes	0.172996	2.89	0.849	2
MacJap	0.0379747	2.936	0.912	2
MiLon	0.172996	2.717	0.8	2
CrInv	0.172996	2.811	0.772	7
OcCer	0.172996	2.974	0.818	7
OcCor	0.172996	2.936	0.825	7
TuCap	0.172996	2.911	0.795	7
TuPol	0.172996	2.935	0.794	7
PiVes	0	2.208	0.618	2

ChGra	0.172996	1.673	0.411	2
CaSap	0.172996	0.746	0.112	2
ChFer	0.172996	0.986	0.158	2
ChaJap	0.172996	1.114	0.179	2
ChNat	0.172996	1.092	0.175	2
MoGla	0.172996	1.032	0.197	2
PoPel	0.172996	0.482	0.04	2
PoSan	0.172996	0.441	0.045	2
PoTri	0.172996	0.435	0.047	2
ScOli	0.172996	1.083	0.188	2
ScPar	0.172996	1.177	0.213	2
ScSer	0.172996	1.234	0.217	2
ScTra	0.172996	1.1	0.217	2
ThCre	0.172996	0.952	0.149	2
GeDeh	0	2.94	0.901	6
HuLic	0	2.978	0.901	6
LoXiu	0.00843882	2.935	0.9	6
LoYan	0.00843882	2.951	0.907	6
PoMot	0	2.76	0.864	6
SiYaa	0.00843882	2.906	0.882	6
LyBre	0.172996	2.913	0.843	2
RaRan	0.172996	2.952	0.859	2
UmOri	0.172996	2.741	0.791	2
AtFlo	0.00421941	2.203	0.591	2
AtInt	0.00421941	2.207	0.592	2
LeSan	0.172996	2.203	0.597	2
